# Image quality assessment of ECG-less coronary CT angiography: A comparative study with conventional ECG-gated CCTA

**DOI:** 10.1016/j.ejro.2026.100794

**Published:** 2026-07-08

**Authors:** Sanaz Asadian, Seyed Ali Nabipoorashrafi, Farbod Khosravi, Alex Diaz, Gulnoor Sheriff, Luis Landeras, Hamid Chalian

**Affiliations:** a Department of Radiology, Cardiothoracic Imaging Section, University of Washington, 1959 NE Pacific Street Room RR215F, Seattle, WA 98195, USA; b Department of Radiology, University of Chicago, 5841 South Maryland Ave, MC206, Chicago, IL 60615, United States

**Keywords:** Coronary Angiography, Computed Tomography Angiography, Electrocardiography, ECG-less CCTA

## Abstract

**Background:**

Electrocardiogram (ECG)-gated coronary CT angiography (CCTA) is a standard method for evaluating coronary artery disease. However, it has limitations, including patient discomfort, technical setup, and reduced accuracy in cases of arrhythmia or obesity. New CT technologies enable high-quality image acquisition without the need for ECG leads.

**Objective:**

This study compares image quality between ECG-less and ECG-gated CCTA.

**Methods:**

We retrospectively analyzed two matched groups of patients (n = 43 each) who underwent either ECG-less or ECG-gated CCTA. ECG-less datasets were retrospectively categorized into subphases corresponding to one-quarter, one-half, three-quarters, and full cardiac cycle reconstructions. Image quality was evaluated by radiologists using both subjective and objective measures for both groups and for all reconstructed datasets. Subjective assessment was performed using a 6-point Likert scale to rate motion artifacts in the left anterior descending artery (LAD), left circumflex artery (LCX), and right coronary artery (RCA). Objective image quality was quantified by calculating the signal-to-noise ratio (SNR) and contrast-to-noise ratio (CNR) in the left main coronary artery (LMCA), LAD, LCX, RCA, and ascending aorta.

**Results:**

Image quality was comparable between the two groups in most subphases. However, in the ¼-cycle subphase in the ECG-less group, Likert scores were significantly lower for the LCX (P = 0.027), and RCA (P = 0.041) compared to the control group. Regarding SNR and CNR, there were no significant differences across all vascular territories in either full-phase or subphase data (P > 0.05).

**Conclusion:**

Both subjective and objective image quality metrics were comparable between ECG-less and conventional ECG-gated CCTA, supporting the reliability of ECG-less acquisitions.

## 1 Introduction

Coronary computed tomography angiography (CCTA) is a first-line diagnostic tool for evaluating coronary artery disease, especially in patients with low to intermediate pre-test probability, as recommended by the American Heart Association (AHA) and the European Society of Cardiology (ESC) [1], [2]. Technically, CCTA is believed to require electrocardiogram (ECG) gating to synchronize image acquisition with the quiescent phases of the cardiac cycle, thereby minimizing motion artifacts and enhancing image quality.

With the expanding use of CCTA in daily clinical practice, imaging departments are experiencing a marked increase in patient volume. This surge places growing demands on specialized staff and complicates appointment scheduling. ECG-gated CCTA, despite its significant diagnostic value, requires patient preparation such as ECG lead placement. This adds to the workload of radiology teams and may pose challenges for patients requiring urgent imaging. As a result, there is a growing interest in implementing streamlined protocols that shorten the procedure and allow for more patients to be accommodated per shift with greater efficiency [3].

Recent innovations in CT hardware and software, such as wide detector coverage, accelerated gantry rotation, and advanced motion correction algorithms, have enabled high-quality CCTA through pulse rate–dependent cardiac cycle simulation and automated phase selection and high-quality reconstruction. These improvements reduce the reliance on ECG lead placement and gating, streamlining the imaging process [4], [5], [6], [7], [8].

Thomsen et al. demonstrated the technical feasibility of performing coronary CT angiography without ECG lead placement using a novel acquisition and reconstruction framework incorporating simulated cardiac cycle information, automated phase selection, and motion-correction algorithms. Although that study has demonstrated the feasibility of CCTA without ECG gating [4], no comparison was performed to assess the image quality of the novel ECG-less protocol in comparison to the traditionally accepted ECG-gated standard protocol. The present investigation was therefore designed as an extension of that work, incorporating a matched ECG-gated control cohort and objective quantitative image quality metrics to enable direct comparison with the current clinical standard.

Importantly, the motivation for investigating ECG-less CCTA is not to replace conventional ECG-gated acquisitions, which remain the current standard for coronary imaging, but rather to evaluate whether recent advances in CT technology can maintain acceptable image quality in the absence of ECG lead placement. Although ECG lead application itself is generally straightforward and requires minimal time, challenges may arise in selected clinical settings, including emergency imaging environments, high-throughput practices, patients with poor electrode adherence, motion-related ECG signal degradation, or situations in which rapid image acquisition is desirable.

In the present investigation, in continuation with the previous studies, ECG-less CCTA image quality was assessed both qualitatively and quantitatively across defined anatomical regions and various subphases of the cardiac cycle. The results were then compared with those obtained from a matched control group of patients who underwent conventional ECG-gated CCTA.

## 2 Methods and materials

This retrospective study received approval from the institutional review board (IRB). In the ECG-less group, informed consent was obtained from all enrolled participants. For the control group, the requirement for informed consent was waived due to the retrospective nature of the study and the fact that these patients had received standard clinical care.

### 2.1 Study subjects

This study included two patient groups who underwent CCTA using either an ECG-less or conventional ECG-gated protocol (control), with 43 patients in each group. All participants in both groups were ≥ 18 years of age. Patients with a history of coronary artery bypass grafting or coronary stent placement were excluded before cohort selection because these prior interventions could influence image quality assessment and quantitative measurements. Following application of exclusion criteria, ECG-less and ECG-gated cohorts were selected. The ECG-less group comprised 43 consecutive individuals scheduled for a clinically indicated CCTA. A control group of 43 patients who underwent standard ECG-gated CCTA was matched at the group level based on age, sex, body mass index (BMI), and heart rate (HR). These parameters were selected because of their known influence on coronary CT image quality and motion-related artifacts. Baseline characteristics of the resulting cohorts were subsequently compared to confirm comparability between groups.

### 2.2 Image acquisition protocol

All scans were performed using a 16 cm wide-coverage CT scanner (Revolution Apex, GE Healthcare). Image acquisition followed a standardized institutional protocol, preceded by HR control using metoprolol for patients with HR exceeding 65 bpm, along with nitroglycerin administration unless contraindicated.

The scan range extended from above the tracheal bifurcation to the base of the heart. Imaging parameters included: Tube voltage: 80–100 kVp or 120 kVp, tube current: automatically modulated, maintained between 150 and 1300 mA, and rotation speed: 0.28 s per rotation. Tube voltage (80–120 kVp) was selected according to institutional clinical practice based on patient body habitus and expected attenuation characteristics. The same tube-voltage selection strategy was applied to both the ECG-less and ECG-gated cohorts. Contrast (Omnipaque [350 mg/mL]) was administered based on the institutional protocol, using a multi-stage method, with a maximum total contrast-saline volume of 150 cc at a rate of 5 cc/sec.

The primary distinction between the study groups was the use of ECG leads and gating. For ECG-less examinations, conventional ECG lead placement was not performed. Instead, cardiac cycle information was estimated using pulse-based physiologic monitoring integrated into the scanner workflow. The pulse signal was used to derive an estimated heart rate, from which a simulated ECG waveform was generated. This simulated waveform enabled operation of the scanner’s standard cardiac reconstruction framework. The resulting reconstruction process was therefore based on estimated cardiac cycle timing rather than direct ECG synchronization [4]. In contrast, for the control group, the standard ECG gating with electrode placement was performed.

All images were reconstructed at a slice thickness of 0.625 mm. For the ECG-less group, full cardiac cycle (full-cycle) image data were further segmented into three one-quarter, one-half, and three-quarter subphases to retrospectively reconstruct image datasets. These reduced reconstruction subphases were derived from the same source acquisition using the vendor-specific reconstruction software. All subphase reconstruction datasets were generated for each patient and subsequently subjected to identical qualitative and quantitative image quality assessments. In both groups, automated phase selection (SmartPhase, GE Healthcare) and motion correction algorithms (SSF2, GE Healthcare) were employed to reduce motion artifacts [4]. Consequently, comparisons were performed between reconstructions generated from different proportions of the available acquisition data rather than between different cardiac phases.

### 2.3 Image analysis

All CCTA examinations were independently interpreted by two cardiothoracic radiologists, each blinded to patient grouping and the other's findings. Readers were instructed to assess each dataset on its own merits and did not use the full-cycle reconstruction as a reference standard during scoring. All image datasets were anonymized prior to review, and readers were blinded to acquisition and reconstruction information, including whether examinations were obtained using ECG-less or conventional ECG-gated techniques. Multiplanar image evaluation was conducted using the Visage Picture Archiving and Communication System (Visage Imaging, Inc., San Diego, CA, USA). Radiologists were free to adjust image orientation and window width/level according to their usual diagnostic workflow.

Assessment was performed for the following anatomic structures: Ascending aorta (AA), left main coronary artery (LMCA), left anterior descending artery (LAD), left circumflex artery (LCX), and right coronary artery (RCA).

Qualitative evaluation included scoring of the motion artifacts at each location using a six-point Likert scale; 0: motion assessment not possible due to poor image quality unrelated to motion, 1: Completely unreadable due to motion; non-diagnostic, 2: Significant motion; limited interpretability, 3: Apparent motion artifact; interpretable, 4: Minor motion artifact; interpretable, 5: No motion artifact; interpretable.

Signal-to-noise ratio (SNR) and contrast-to-noise ratio (CNR) were calculated for five vascular sites. Measurements were taken in the mid-AA and proximal segments of the four coronary arteries, selecting the best-visualized portion where a circular region of interest (ROI) could be delineated within the vascular lumen. A matching-diameter ROI was placed in adjacent perivascular fat to serve as a background noise reference.

ROIs were consistently drawn to exclude calcified areas, ensuring accurate attenuation measurements. The mean intraluminal Hounsfield Unit (HU) value was recorded, and quantitative indices were calculated as follows:

SNR = Mean intraluminal HU / SD of perivascular fat HU

CNR = (Mean intraluminal HU − Mean perivascular fat HU) / SD of perivascular fat HU.

For the ECG-less group, all indices were measured at full-cycle series as well as ¼, ½, and ¾ subphases.

A final consensus was reached for both qualitative and quantitative assessments prior to including the data in the analysis. In addition to image quality assessment, coronary artery disease severity was evaluated using the coronary artery disease–reporting and data system (CAD-RADS) classification.

### 2.4 Statistical analysis

Continuous variables were reported as mean ± SD or median [Q1–Q3], and categorical variables were presented as counts and percentages. The Kolmogorov–Smirnov test was used to assess normality of the distribution. Comparisons of numerical data were performed using the Mann–Whitney *U* test, while categorical variables were compared using the chi-square test. For the final analysis, qualitative and quantitative measures were averaged across the full-cycle as well as ¼, ½, and ¾ subphases for the ECG-less group which consequently were compared with the findings of the matched control group. Interobserver agreement was assessed using weighted kappa for ordinal Likert scale ratings and intraclass correlation coefficient (ICC) for continuous variables, specifically SNR and CNR. All analyses were performed using SPSS version 26 (IBM Corp., Armonk, NY, USA), and a two-tailed p-value < 0.05 was considered statistically significant.

## 3 Results

A total of 86 patients (43 in each group) were enrolled in this study, after application of the exclusion criteria and completion of cohort matching, with 22 males (51.2%) in each group. The mean ± SD age, BMI, and HR were 62 ± 12, 28.6 ± 6.8, and 65.3 ± 9.0 for the ECG-less group, and 58 ± 13.1, 29.73 ± 7.4, and 67 ± 9.3 for the control group, with no significant differences (Table 1 and Supplementary Table S1). Also, no statistically significant difference was observed in CAD-RADS scores between the ECG-less and ECG-gated groups (P > 0.05).

**Table 1:** Baseline characteristics of the study groups.

Characteristic	Case (n = 43)	Control (n = 43)	P
Female sex, %	21 (48.8%)	21 (48.8%)	1.000
Age,y, mean ± SD	62 ± 12.2	58 ± 13.1	0.071
BMI (kg/m^2^), mean ± SD	28.6 ± 6.8	29.73 ± 7.4	0.739
Heart Rate (beats/min), mean ± SD	65.3 ± 9.0	67.2 ± 9.3	0.938
Beta blockers	21 (48.8%)	23 (53.4%)	0.666
Nitroglycerin	34 (79.1%)	32 (74.4%)	0.610
CAD-RADS (median, IQR)	3 (2)	4 (4)	0.092

BMI: Body Mass Index; IQR: Interquartile range; CAD-RADS: Coronary Artery Disease - Reporting and Data System.

The mean dose-length product (DLP) for the ECG-less group was 679.4 ± 282.1 mGy·cm, compared with 663.4 ± 401.2 mGy·cm for the ECG-gated control group, with no significant difference observed between groups (P = 0.85).

### 3.1 Interobserver variability studies

For evaluation of interobserver variability in qualitative assessment, Kappa coefficients were calculated. The weighted Kappa values ranged from 0.72 to 0.78 across various anatomic locations, indicating substantial interobserver agreement. For evaluation of interobserver variability in quantitative assessment, the ICC was calculated, ranging from 0.79 to 0.84 across different anatomic locations, indicating good to excellent interobserver agreement.

### 3.2 Image quality analysis

#### 3.2.1 Qualitative assessment

**Fig. 1:**
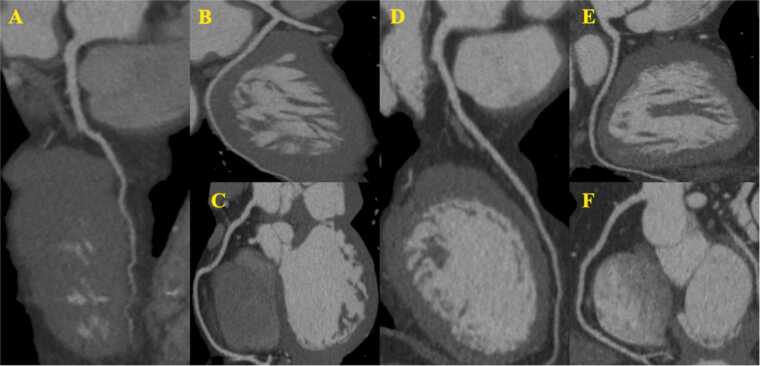
Curved multiplanar reconstructed images of coronary arteries of a patient who underwent standard electrocardiogram-gated CT angiography (A-C) and a matched patient from the electrocardiogram-less group in whom ¾ of the image data was used (D-F). The quality of images could be compared appropriately. A and D: Left anterior descending artery, B and E: Left circumflex artery, C and F: Right coronary artery.

The median [Q1-Q3] Likert scores in the control group were 3 [2.56–3.00] for the LAD, 3 [3.00–3.00] for the LCX, and 3 [3.00–3.00] for the RCA (Table 2). Comparison of the corresponding Likert scores for the full-cycle as well as ½ and ¾ subphases with the control group demonstrated no significant differences (P > 0.05) (Fig. 1). However, when comparing the Likert score values from the ¼ subphase image series with those of the control group, a significant difference was found for the LCX and RCA, with P = 0.027 and P = 0.041, respectively. For LAD, no significant difference was observed between the ¼ subphase image dataset and the control group (P > 0.05). Although statistically significant differences in Likert scores were observed for the LCX and RCA in the ¼-cycle subphase reconstruction compared with the ECG-gated control group, these findings should be interpreted with caution. Given the exploratory nature of the study and the multiple comparisons performed across coronary territories and reconstruction subphases, formal correction for multiple testing was not applied. Therefore, the possibility of type I error cannot be excluded, and these observations should be considered hypothesis-generating rather than definitive evidence of reduced image quality.

**Table 2:** Subjective image quality assessment.

Vessels	LCX	P	LAD	P	RCA	P
Reference median [Q1–Q3]	3 [3.00–3.00]	ref	3 [2.56–3.00]	ref	3 [3.00–3.00]	ref
1/4 median [Q1–Q3]	3 [2.00–3.00]	0.027*	3 [3.00–3.00]	0.247	3 [2.00–3.00]	0.041*
2/4 median [Q1–Q3]	3 [3.00–3.00]	0.416	3 [3.00–3.00]	0.115	3 [3.00–3.00]	0.860
¾ median [Q1–Q3]	3 [2.00–3.00]	0.091	3 [3.00–3.00]	0.222	3 [2.00–3.00]	0.438
4/4 median [Q1–Q3]	3 [2.00–3.00]	0.091	3 [3.00–3.00]	0.200	3 [2.00–3.00]	0.208

LAD: Left Anterior Descending Artery; LCX: Left Circumflex Artery; RCA: Right Coronary Artery;.

#### 3.2.2 Quantitative assessment

**Fig. 2:**
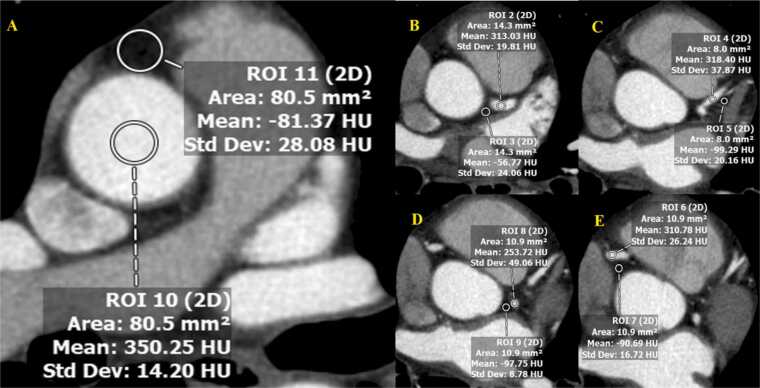
Axial coronary computed tomography scans demonstrate the five anatomic locations on which the signal-to-noise ratio and contrast-to-noise ratio were measured. A. mid-ascending aorta, B. Left main coronary artery, C. Left anterior descending artery, D. Left circumflex artery, and E. Right coronary artery.

SNR and CNR for the control group in the AA, LMCA, LAD, LCX, and RCA, were compared to the ECG-less group across the full-cycle and the three subphases (Fig. 2). No significant differences were observed between the ECG-less group (in either the full-cycle or subphases) and the control group (P > 0.05) across all five locations (Table 3) (Supplementary Figure S1).

**Table 3:** Objective Image Quality Assessment.

LMCA: Left Main Coronary Artery; LAD: Left Anterior Descending Artery; LCX: Left Circumflex Artery; RCA: Right Coronary Artery; CNR: Contrast-to-Noise Ratio; SNR: Signal-to-Noise Ratio;.

## 4 Discussion

In this study, the image quality of a novel ECG-less CCTA protocol was compared with that of conventional ECG-gated CCTA. Both qualitative assessment using a Likert scale and quantitative evaluations of CNR and SNR were performed across key anatomic regions. Results demonstrated that the ECG-less CCTA protocol yielded no significant differences in image quality across all quantitative and qualitative indices in the full-cycle, as well as in the ½ and ¾ subphases, compared to the standard ECG-gated approach. Similarly, in the ¼ subphase, no significant differences were observed for quantitative metrics and most qualitative scores, except for the Likert ratings of the LCX and RCA.

The clinical demand for CCTA is steadily increasing, particularly among patients with low to intermediate risk of coronary artery disease [9]. In response, both healthcare providers and imaging technology developers are actively seeking faster and more efficient scanning protocols to enhance workflow and broaden accessibility.

Compelling evidence for ECG-less CCTA is provided by a recent investigation by Thomsen et al., who introduced a novel protocol that simulates the ECG waveform and cardiac cycle based on estimated HR rather than using conventional ECG leads [4]. Their approach leveraged automated phase selection and innovative motion correction algorithms to optimize image reconstruction. The study demonstrated that acceptable coronary image quality could be achieved using the full-cycle dataset, and notably, image quality remained viable when only ¾ or ½ of the acquired data was utilized. A significant decline in image quality was observed only when comparing full-cycle reconstructions to the ¼ subphase across most HR categories. Although the study did not include a control group, it established the feasibility of ECG-less CCTA and proposed a framework for developing rapid imaging protocols that may not require full-cycle data, particularly beneficial for streamlined coronary evaluation in urgent care settings. We view the present study as both an extension and a validation of the feasibility work by Thomsen et al. Building on their initial findings, our study adds value by directly comparing results with a matched ECG-gated cohort and by incorporating objective, quantitative measures of image quality. This approach allows for a more robust and comprehensive assessment of the technique’s performance.

In a separate investigation, Wang et al. evaluated an ECG-less CCTA protocol for the detection of coronary plaques and stenosis [3]. Like our findings, their study demonstrated that the image quality is comparable to that of conventional ECG-gated CCTA, though the evaluation was limited to a full-cycle acquisition. They emphasized that while this approach offered reduced acquisition time, it was associated with significantly increased radiation exposure. While our results were in line with those of Wang et al., we also explored whether image quality could be maintained when reconstructions were generated from reduced portions of the available acquisition data. Although this approach may have implications for future acquisition optimization strategies, the present study was not designed to evaluate radiation dose reduction.

Based on our findings, qualitative image quality ratings were comparable to those of standard ECG-gated CCTA across all evaluated anatomical regions and subphases, except for the RCA and LCX when only one-quarter of the data was used for image reconstruction. This discrepancy can be attributed to the rapid systolic motion of the proximal RCA, which leads to contour blurring. Additionally, the mid-RCA is perpendicular to the scan plane, resulting in pronounced in-plane motion artifacts. The LCX, by contrast, occupies a posterior location and follows a looped trajectory, both of which contribute to partial volume effects and motion-related distortions. It is widely recognized that even with standard ECG-gated CCTA protocols, the RCA, followed by the LCX, is more susceptible to motion artifacts and blurring compared to more stable segments such as the LAD and LMCA [10], [11]. We postulate that this susceptibility may be more pronounced when image reconstruction is limited to reduced-cycle data and the optimal cardiac phase is not captured within the available acquisition window. An additional consideration when interpreting these findings is the large number of statistical comparisons performed across multiple coronary vessels and reconstruction subphases. As this study was designed as an exploratory image-quality investigation, no formal adjustment for multiple testing was applied. Consequently, the statistically significant differences observed in the RCA and LCX during the ¼-cycle subphase reconstruction may represent true differences in subjective image quality, but they may also reflect chance findings related to multiple comparisons. Nevertheless, the fact that these differences occurred in vascular territories known to be particularly susceptible to cardiac motion provides physiologic plausibility for the observations. Future studies with larger cohorts and pre-specified statistical adjustment strategies are needed to confirm whether these findings represent clinically meaningful limitations of aggressive data reduction approaches.

On the other hand, the lower subjective image quality observed in the RCA and LCX using the ¼-cycle reconstruction subphase should not be interpreted as evidence of non-diagnostic image quality. Although image quality scores were significantly lower than those of the ECG-gated control group, the median ratings remained within the diagnostically interpretable range (Likert score: 3 for both vessels). These findings suggest that aggressive reduction of reconstruction data may increase susceptibility to motion-related image degradation in certain coronary segments, particularly the RCA and LCX, while still preserving overall interpretability in most cases.

Assuming that the image quality may be influenced by the presence of coronary plaque, calcification, and stenosis, the distribution of CAD-RADS categories was evaluated in both cohorts. The study population included a range of disease severities extending beyond normal coronary arteries, supporting the applicability of the findings to a broader clinical population. Nevertheless, the present investigation was designed to assess image quality rather than diagnostic accuracy, and future studies should specifically evaluate the performance of ECG-less CCTA across different CAD-RADS categories and lesion characteristics.

Another finding of our investigation is that the quantitative quality measures, SNR and CNR, were comparable to those of the standard ECG-gated protocol in all anatomic locations and for all included series. The modest discrepancy between qualitative and quantitative assessments is describable by the fact that qualitative assessments reflect subjective human perception, which can be influenced by subtle visual cues like motion blur, edge sharpness, or anatomical context, while quantitative metrics are objective, focusing on pixel-level statistics that may not fully capture perceptual distortions.

It is noteworthy that the present findings should not be interpreted as suggesting that ECG-less CCTA should replace conventional ECG-gated CCTA in routine clinical practice. ECG gating remains the established standard for CCTA and provides reliable synchronization of image acquisition with the cardiac cycle. Rather, the objective of this study was to determine whether modern CT acquisition and reconstruction technologies can preserve image quality when conventional ECG-based synchronization is not available or is impractical. The comparable image quality observed in most evaluated datasets suggests that ECG-less approaches may have potential utility in selected circumstances, such as workflow-constrained environments, urgent imaging settings, or situations in which ECG acquisition is technically challenging. Nevertheless, image quality equivalence does not necessarily imply diagnostic equivalence, and further studies assessing stenosis detection, plaque characterization, diagnostic accuracy, and clinical outcomes are required before broader clinical implementation can be considered.

## 5 Limitations

Despite the valuable findings, this study has several limitations. First, the case and control groups consisted of different patient populations. Although careful matching was performed, inherent anthropometric differences between the groups may still have influenced the results. Ideally, in a prospective study, both imaging protocols should be assessed within the same patient population to ensure direct comparability. However, implementing such a study raises ethical concerns due to the additional radiation exposure that duplicate scans would entail. Another limitation of this study is the relatively small sample size, which may have limited the statistical power to detect subtle but potentially clinically meaningful differences between ECG-less and conventional ECG-gated CCTA. Consequently, the absence of statistically significant differences in several comparisons should not be interpreted as definitive evidence of equivalence. Rather, the present study should be considered an exploratory validation investigation focused on image quality assessment. Larger prospective studies with adequately powered cohorts, ideally conducted across multiple centers and encompassing a broader spectrum of coronary artery disease severity, are warranted to confirm the reproducibility and generalizability of these findings.

Another limitation of this study is that all examinations were performed using a single CT platform (GE Revolution Apex) and relied on vendor-specific technologies, including SmartPhase automated phase selection and SSF2 motion-correction algorithms. Consequently, the observed image quality performance may reflect, in part, characteristics of this specific acquisition and reconstruction framework. The extent to which these findings can be generalized to other CT vendors, scanner generations, or reconstruction algorithms remains uncertain. Future multicenter studies incorporating multiple imaging platforms will be necessary to determine the broader applicability of ECG-less CCTA across different technological environments.

The ¼-, ½-, and ¾-cycle reconstruction subphases were derived from the same source acquisition. Thus, the resulting datasets were not fully independent observations. Consequently, comparisons among reconstruction subphases should be interpreted as exploratory evaluations of reconstruction performance rather than as comparisons of independent acquisition strategies. Moreover, in this study, the individual contributions of the SmartPhase automated phase-selection software and the SSF2 motion-correction algorithm were not evaluated separately. Both tools were incorporated into the standard reconstruction workflow for all examinations and were therefore assessed only as part of the overall imaging protocol. Consequently, the extent to which image quality preservation in ECG-less CCTA was attributable to automated phase selection, motion correction, or other aspects of the acquisition and reconstruction process cannot be determined from the current data. Future studies specifically designed to compare reconstruction strategies with and without these algorithms may help clarify their respective contributions.

It should be mentioned that the present study did not systematically evaluate pulse-rate variability during image acquisition. Although acquisition times were short and automated phase-selection and motion-correction algorithms were applied to compensate for minor physiologic variability, the effect of larger fluctuations in pulse-derived heart rate on ECG-less image quality remains to be determined.

We believe that although image datasets were anonymized and readers were blinded to acquisition and reconstruction information, complete blinding may not have been achievable because certain reconstruction characteristics could potentially be recognized by experienced readers. Consequently, the possibility of residual observer bias cannot be entirely excluded. It is noteworthy that the interobserver agreement observed in this study was substantial for qualitative assessments and good for quantitative measurements, supporting the reproducibility of the image quality evaluations. Nevertheless, the inherent subjectivity of visual image assessment may contribute to residual variability, and future studies incorporating larger reader panels may further improve assessment robustness.

Finally, an important limitation of this study is the absence of an external diagnostic reference standard. No comparison with invasive coronary angiography, fractional flow reserve, or other independent reference standards was performed in this investigation. Consequently, although image quality was comparable between ECG-less and conventional ECG-gated CCTA in most analyses, the extent to which these findings translate into equivalent diagnostic performance for coronary stenosis assessment or plaque characterization remains unknown. Future studies incorporating lesion-level analyses and external diagnostic reference standards are needed to determine whether image quality equivalence translates into diagnostic equivalence.

## 6 Conclusion

ECG-less CCTA demonstrated image quality comparable to conventional ECG-gated CCTA for full-cycle and most partial-cycle reconstructions. However, reduced subjective image quality observed in the RCA and LCX using the ¼-cycle reconstruction suggests caution when aggressive data reduction strategies are considered. These findings support the technical feasibility of ECG-less CCTA and warrant further investigation of its diagnostic performance across diverse clinical populations and coronary disease severities.

## Statement of studies in humans and animals

Ethical approval was obtained from the Institutional Review Board (IRB) of the University of Washington, Seattle, WA, USA.

This study was conducted in accordance with relevant guidelines and regulations and in compliance with the Declaration of Helsinki.

The requirement for informed consent was waived due to the retrospective nature of the study.

No animals were involved in this study.

## Key finding

*ECG-less coronary CT angiography (CCTA) demonstrated comparable image quality to standard ECG-gated CCTA in both subjective and objective measures.

*Quantitative metrics, including signal-to-noise ratio (SNR) and contrast-to-noise ratio (CNR), showed no significant differences between ECG-less and ECG-gated CCTA across all cardiac phases and vascular territories.

## Importance summary

The study evaluated a novel ECG-less CCTA protocol and found it effective across multiple image quality measures when compared to the standard ECG-gated approach. These results highlight its potential for broader adoption in clinical workflows without compromising diagnostic performance.

## Funding

This project did not receive any funding from any organization or institute.

## CRediT authorship contribution statement

**Sanaz Asadian:** Writing – review & editing, Writing – original draft, Investigation, Formal analysis, Data curation, Conceptualization. **Seyed Ali Nabipoorashrafi:** Writing – review & editing, Writing – original draft, Software, Methodology, Investigation, Conceptualization. **Farbod Khosravi:** Writing – review & editing, Writing – original draft. **Alex Diaz:** Writing – review & editing, Software, Investigation. **Gulnoor Sheriff:** Writing – review & editing, Resources. **Luis Landeras:** Writing – review & editing, Conceptualization. **Hamid Chalian:** Writing – review & editing, Methodology, Investigation, Conceptualization.

## Declaration of Competing Interest

Hamid Chalian and Seyed Ali Nabipoorashrafi are recipients of the GE HealthCare and NASCI Research Grant. All other authors have nothing to declare.
