# Supplementary material for: Image quality assessment of ECG-less coronary CT angiography: A comparative study with conventional ECG-gated CCTA
Source: Eur J Radiol Open. 2026 Jul 8;17:100794. doi: 10.1016/j.ejro.2026.100794 (PMC13379992; doi:10.1016/j.ejro.2026.100794)
Supplement: Supplementary file 1 — Supplementary material [file mmc1.docx]

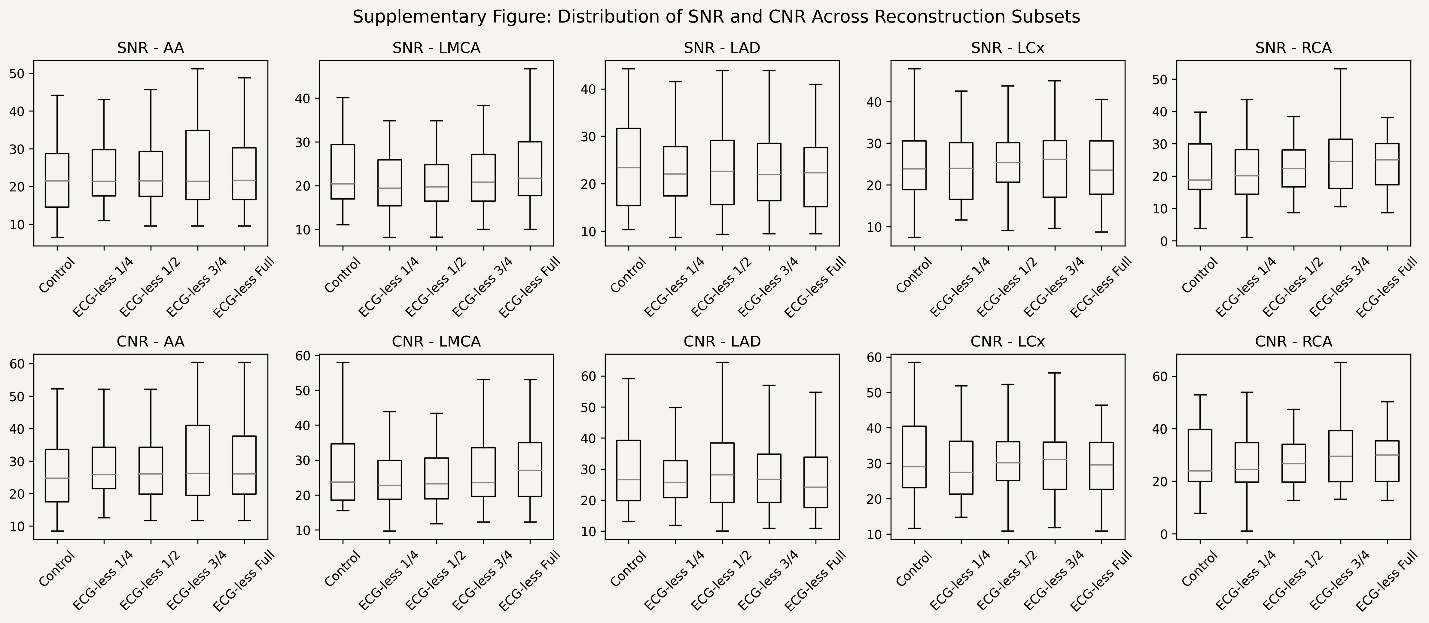
Supplementary Figure S1. Boxplot distribution of signal-to-noise ratio (SNR) and contrast-to-noise ratio (CNR) measurements across the ascending aorta (AA), left main coronary artery (LMCA), left anterior descending artery (LAD), left circumflex artery (LCX), and right coronary artery (RCA). Quantitative image quality metrics are shown for the ECG-gated control group and ECG-less reconstruction subphases (¼-cycle, ½-cycle, ¾-cycle, and full-cycle). The substantial overlap of distributions is consistent with the absence of significant differences in quantitative image quality measurements.
