# Supplementary material for: Image quality assessment of ECG-less coronary CT angiography: A comparative study with conventional ECG-gated CCTA
Source: Eur J Radiol Open. 2026 Jul 8;17:100794. doi: 10.1016/j.ejro.2026.100794 (PMC13379992; doi:10.1016/j.ejro.2026.100794)
Supplement: Supplementary file 2 — Supplementary material [file mmc2.docx]

| Supplementary Table S1. Distribution of CAD-RADS Categories in the ECG-less and ECG-Gated Study Cohorts | | |
| --- | --- | --- |
| CAD-RADS category | ECG-less | Control |
| 0 | 16/43 (37.2%) | 20/43 (46.5%) |
| 1 | 7/43 (16.3%) | 10/43 (23.3%) |
| 2 | 9/43 (20.9%) | 6/43 (14.0%) |
| 3 | 4/43 (9.3%) | 2/43 (4.6%) |
| 4 | 5/43 (11.6%) | 4/43 (9.3%) |
| 5 | 2/43 (4.6%) | 1/43 (2.3%) |
| CAD-RADS: Coronary Artery Disease - Reporting and Data System; ECG: Electrocardiogram | | |
